# Application of a New Thermal Model for the Determination of London Dispersive Properties of H-β-Zeolite/Rhodium Catalysts Using New 2D Chromatographic Models

**DOI:** 10.3390/ma18010081

**Published:** 2024-12-28

**Authors:** Tayssir Hamieh

**Affiliations:** 1 Faculty of Science and Engineering, Maastricht University, P.O. Box 616, 6200 MD Maastricht, The Netherlands; t.hamieh@maastrichtuniversity.nl; 2 Institut de Science des Matériaux de Mulhouse, Université de Haute-Alsace, CNRS, IS2M UMR 7361, F-68100 Mulhouse, France; 3 Laboratory of Materials, Catalysis, Environment and Analytical Methods Laboratory (MCEMA), Faculty of Sciences, Lebanese University, Hadath P.O. Box 6573, Lebanon

**Keywords:** Hamieh thermal model, London dispersive surface energy, catalyst, adhesion work, enthalpy and entropy of adhesion

## Abstract

A new methodology based on the Hamieh thermal model was applied for the determination of the surface properties of solid surfaces. The new approach consisted of the accurate quantification of the London dispersive surface energy of materials using the two-dimensional inverse gas chromatography technique at infinite dilution. This technique used the notion of the net retention volume of adsorbed molecules on the solid catalysts, allowing the determination of the free energy of adsorption. The Hamieh thermal model proving the temperature effect on the surface area of organic molecules adsorbed on H-β-zeolite/rhodium catalysts at different rhodium percentages was used to determine the accurate values of the London dispersive surface energy of solid surfaces at different temperatures. This new method also allowed a precise evaluation of the dispersive adhesion work, dispersive surface enthalpy, and entropy of adsorption of n-alkanes adsorbed on the catalysts. In this paper, the London dispersive surface energy and adhesion work of H-β-zeolite-supported rhodium catalysts were determined using the free energy of adsorbed molecules obtained from the two-dimensional inverse gas chromatography technique at infinite dilution. It was proved that the London dispersive surface energy strongly depended on the temperature and the rhodium percentage, while the dispersive adhesion work of n-alkanes adsorbed on H-β-zeolite/rhodium catalysts was proved to be a function of the temperature, rhodium percentage, and the carbon atom number of the n-alkanes.

## 1. Introduction

A two-dimensional (2D) inverse gas chromatography (IGC) technique at infinite dilution [1,2,3,4,5,6,7,8,9,10,11,12,13,14,15,16,17,18,19,20,21,22,23,24,25,26,27,28,29] has been widely used for the surface characterization of solid materials such as oxides [8,13,14,16,18,26], metals [26,27,28,29], polymers [2,3,5,17,18], fibers [5,6,21,22], biomaterials [14,15,26] and nanomaterials, ceramics, catalysts [12,13,19,23,24,25,26,27,28,29], and pharmaceutical [9,11,15] and food products [9,10]. The injection of infinitesimal quantities of gas molecules into a chromatographic column filled by a solid substrate will realize an adsorption of these molecules on the solid surface and can be described by the two-dimensional state of gas, relating its surface pressure to the surface area of the solid material. The chromatographic measurements lead to the net retention volume $V_{n}$ of the organic solvents adsorbed on the solid surfaces. Considering that the adsorption of gas molecules is in reversible equilibrium on a solid substrate, it is possible to obtain the following standard free energy $∆G_{a}^{0}$ of adsorption of a molecule:

(1) $$∆G_{a}^{0}=-RT\;ln\;V_{n}+RT\;ln\;\left(\frac{{sm\pi }_{0}}{P_{0}}\right)$$

where *R* is the perfect gas constant, *T* the absolute temperature, *m* the mass of the solid introduced in the chromatographic column, *s* the specific surface area, and $P_{0}$ and $\pi_{0}$ represent the reference states, respectively, of pressure and two-dimensional pressure.

The IGC technique at infinite dilution was proved to be the most interesting surface technique to investigate the thermodynamic surface properties of solid materials [1,2,3,4,5,6,7,8,9,10,11,12,13,14,15,16,17,18,19,20,21,22,23,24,25,26,27,28,29,30,31,32].

The net retention volume considered as a fundamental thermodynamic parameter allows one to obtain the dispersive free energy $\left(-∆G_{a}^{0}\right)$, the London dispersive surface energy $\gamma_{s}^{d}$, the polar enthalpy $\left(-∆H_{a}^{p}\right)$ and entropy $\left(-∆S_{a}^{p}\right)$ of adsorption, and the Lewis acid-base properties of materials.

The surface physicochemical properties of solid materials or nanomaterials were studied by several scientists using the IGC technique at infinite dilution [1,2,3,4,5,6,7,8,9,10,11,12,13,14,15,16,17,18,19,20,21,22,23,24,25,26,27,28,29]. The determination of these surface properties is of vital interest in many industrial processes such as catalysis, adhesion, chemical engineering, colloidal dispersions, and other industrial applications. The IGC methods [21,22,23,24,25,26] and molecular models [27,30,31,32] were applied in the literature to determine the surface energetic properties of solid particles. The experimental determination of the net retention volume was utilized for the calculation of the dispersive $\left(-∆G_{a}^{d}\right)$ and polar $\left(-∆G_{a}^{p}\right)$ free energies of organic solvents adsorbed on solid materials using the following thermodynamic equation:

(2) $$\left(-∆G_{a}^{0}\right)=RTln\;V_{n}+C\left(T\right)=\left(-∆G_{a}^{d}\right)+\left(-∆G_{a}^{p}\right)$$

where *R* is the perfect gas constant, T the absolute temperature, and C(T) a constant parameter depending on T and the interaction between the solid materials and the organic molecules.

The first method used to calculate the London dispersive surface energy of materials was based on Dorris and Gray [33] and the well-known relationship of Fowkes [34] relating the dispersive work of adhesion $W_{a}^{d}$ to the free dispersive energy of adsorption $∆G_{a}^{d}$ by Equation (3):

(3) $$\left(-∆G_{a}^{d}\right)=Na\;W_{a}^{d}=2Na\;\sqrt{\gamma_{l}^{d}\gamma_{s}^{d}}$$

where N is Avogadro’s number, a the surface area of adsorbed solvent, and $\gamma_{l}^{d}$ and $\gamma_{s}^{d}$ are, respectively, the dispersive components of the liquid solvent and the solid.

Dorris and Gray were the first to determine the dispersive component of the surface energy of a solid by defining the increment $∆G_{-CH2-}^{0}$ of the methylene group:

$$∆G_{-CH2-}^{0}=∆G^{0}\left(C_{n+1}H_{2(n+2)}\right)-∆G^{0}\left(C_{n}H_{2(n+1)}\right)$$

where $C_{n}H_{2(n+1)}$ and $C_{n}H_{2(n+1)}$ represent the general formulas of two consecutive n-alkanes.

The London dispersive surface energy $\gamma_{s}^{d}$ of a the solid can therefore be determined by Equation (4):

(4) $$\gamma_{s}^{d}=\frac{{\left[RTln\left[\frac{V_{n}\left(C_{n+1}H_{2(n+2)}\right)}{V_{n}\left(C_{n}H_{2(n+1)}\right)}\right]\right]}^{2}}{4N^{2\;}a_{-CH2-}^{2}\gamma_{-CH2-}}$$

where $a_{-CH2-}$ *is* the surface area of the methylene group taken equal to 6 Å^2^ with a surface energy given by Equation (5):

(5) $$\gamma_{-CH2-}\left(mJ/m^{2}\right)=52.603-0.058T\;\left(K\right)$$

$\gamma_{s}^{d}$ was also determined by a similar method proposed by Schultz et al. representing the variations of $RTln\;V_{n}$ as a function of $2Na\;\sqrt{\gamma_{l}^{d}}$ of the adsorbed n-alkanes using Equation (6):

(6) $$RTln\;V_{n}=2Na\;\sqrt{\gamma_{l}^{d}\gamma_{s}^{d}}+A(T)$$

where A(T) is a constant depending on the temperature. A straight line is obtained, and its slope is equal to $\sqrt{\gamma_{s}^{d}}$.

In a previous paper [35], we criticized the above methods and proposed a new thermal model proving the temperature effect of the surface area a(T) and the London dispersive surface energy of the organic solvents $\gamma_{l}^{d}(T)$. Indeed, the method proposed by Schultz et al. was proved to be inaccurate and cannot be then used to characterize the solid surfaces and obtain quantitative properties, because the authors supposed the surface area and $\gamma_{l}^{d}$ of probes as constant independent from the temperature, while our results proved an important effect of temperature on the values of a(T) and $\gamma_{l}^{d}$ of organic molecules. Furthermore, the Dorris–Gray relation used a constant surface area of the methylene group equal to $a_{-CH2-}=6{Å}^{2}$, whereas, we proved the non-validity of this hypothesis by showing that $a_{-CH2-}\left(T\right)$ depends on temperature. The use of the Hamieh thermal model [35,36] led to more accurate values of the London dispersive surface energy of solid surfaces.

Furthermore, different other methods were applied in the literature to determine the polar variables of adsorption and the Lewis acid-base parameters of solids. These chromatographic methods used different thermodynamic parameters such as the London dispersive surface energy, vapor pressure, boiling point, and polarizability of organic molecules. All these methods used the linear properties of the thermodynamic parameters of the organic solvents. A general linear equation relative to the adsorption of n-alkanes on solid surfaces was previously proposed [34,36]:

(7) $$RTlnV_{n}(T)=\alpha_{j}x_{j}+\beta_{j}$$

where $x_{j}$ represents a surface chromatographic parameter, and $\alpha_{j}$ and $\beta_{j}$ are two constants relative to the adsorption of n-alkanes on the solid surfaces experimentally determined at each temperature from the slope and the ordinate at the origin of the n-alkanes straight line, thus leading to the polar free energy, enthalpy, and entropy of adsorption. The reference parameter $x_{j}$ can be taken from the following variables: the boiling point $T_{B.P.}$ of the solvent proposed by Sawyer and Brookman [20], the vapor pressure $P_{0}$ of the solvent, ${lnP}_{0}$ used by Saint-Flour and Papirer [21,22], the London dispersive surface energy $\gamma_{l\;}^{L}$ of the solvent considered by Schultz et al. [37], the deformation polarizability $\alpha_{0}$ of the organic molecule suggested by Donnet et al. [23], the topological index $\chi_{T}$ determined by Brendlé and Papirer [24,25], and the Hamieh thermal model ${a\left(T\right)\gamma }_{l\;}^{L}(T)$ using the temperature effect on the surface area and the London dispersive surface energy of organic molecules [35,36].

The dispersive adhesion work $W_{a}^{d}(T)$ of n-alkanes on H-β-zeolite/rhodium catalysts as a function of temperature given by Equation (8)

(8) $$W_{a}^{d}(T)=2\;\sqrt{\gamma_{l}^{d}\gamma_{s}^{d}}$$

was never studied in the literature.

In this paper, we applied the new Hamieh thermal model to determine the effect of temperature on the London dispersive surface energy and dispersive adhesion work of H-β-zeolite and the rhodium impregnated in H-β-zeolite at different rhodium percentages. The new results of the London dispersive properties of the different catalysts corrected the previous results [35] by showing a larger deviation percentage between the values of $\gamma_{s}^{d}$ of zeolites obtained using the molecular models and those obtained by the new thermal model. Furthermore, this new study highlighted the effect of the temperature and the rhodium percentage on the dispersive work of adhesion of n-alkanes on the zeolite catalysts.

## 2. Materials and Methods

### 2.1. Materials and Solvents

The rhodium/H-β-zeolites were synthetized in a previous paper [32] using the method developed by Navio et al. [38] and Zhang et al. [39]. The organic solvents utilized for chromatographic measurements obtained from Aldrich (Paris, France) were of high purity grade (i.e., 99%). Several solvents were used, like n-pentane, n-hexane, n-heptane, n-octane, n-nonane, methanol, acetone, trichloroethylene, tetrachloroethylene, diethyl ether and benzene (weak base), chloroform, and cyclohexane (weak acid). The polar organic solvents were characterized by the corrected electron acceptor *AN’* and donor *DN’* numbers determined by Gutmann [26], corrected by Riddle and Fowkes [40], and previously normalized in previous works [26,41].

### 2.2. Experimental

The chromatographic measurements were carried out on a commercial Focus GC gas chromatograph (from Sigma-Aldrich, St. Quentin Fallavier, France) equipped with a flame ionization detector. Dried nitrogen was the carrier gas. The gas flow rate was sustained at 20 mL/min. The temperatures of the injector and detector were maintained at 450 K during the chromatographic measurements [42]. An amount of 0.1 μL of each probe vapor was injected with 1 μL Hamilton syringes to realize the infinite dilution. The columns were prepared using a stainless steel column with a 2 mm inner diameter and with an approximate length of 20 cm. Each column was packed with 1 g of solid particles with a size not exceeding 250 μm. The temperature of the columns varied from 300 K to 430 K. Each probe injection was repeated three times, and the average retention time, *t_R_*, was used for the calculation. The standard deviation of the measurements did not exceed 1%.

The surface specific area of the various catalysts was determined in a previous study by using Brunauer–Emmett–Teller (BET) [32]. The nitrogen adsorption–desorption experiments were carried out using the BET gas adsorption method at 77 K, in an automatic Micromeritics ASAP 2420 apparatus. The obtained specific surface area $S_{BET}$ (defined as the total surface area of a solid material per unit mass, $m^{2}/g$) and the microporous volume *V_m_* of the various catalysts at different rhodium percentages are given in Table 1.

### 2.3. Retention Volume

The most important experimental parameter derived from chromatographic measurements was the net retention volume $V_{n}$ obtained from Equation (9):

(9) $$V_{n}=jD_{c}(t_{R}-t_{0})$$

where $t_{R}$ is the retention time of the adsorbed solvent, $t_{0}$ the zero-retention reference time measured with a non-adsorbing probe such as methane, $D_{c}$ the corrected flow rate, and j a correction factor taking into account the gas compression [42]. The factors $D_{c}$ and j were respectively given by the following relations

(10) $$D_{c}=D_{m}\frac{T}{T_{a}}\;\frac{\eta \left(T\right)}{\eta \left(T_{a}\right)}$$

and

(11) $$j=\frac{3}{2}\;\frac{{\left(\frac{\Delta P+P_{0}}{P_{0}}\right)}^{2}-1}{{\left(\frac{\Delta P+P_{0}}{P_{0}}\right)}^{3}-1}$$

where $D_{m}$ is the measured flow rate (cm^3^/min), T the column temperature (K), $T_{a}$ the room temperature (K), η the gas viscosity (cp), $P_{0}$ the atmospheric pressure (Pa), and ΔP the pressure variation (Pa).

### 2.4. London Dispersive Surface Energy of Catalysts Using the Hamieh Thermal Model

The determined values of London dispersive surface energy $\gamma_{s}^{d}$ and the polar free energy of sold particles at different temperatures strongly depend on the chosen molecular model [17,26,27], and the difference between the values varied and sometimes reached a four times variation from one molecular model to another [26,27,28,29]. In fact, the surface area of organic solvents such as n-alkanes and polar molecules depends on the temperature, while the molecular models give constant values of the surface area [32]. This will lead to wrong values of the surface thermodynamic properties of materials [26,27,28,29,35]. The correction of the above properties can be made by applying the Hamieh thermal model [35]. Indeed, in a recent study, we proved the temperature effect on the surface areas of molecules and proposed the following relation of the surface area $a\left(n,T\right)$ of n-alkanes as a function of the temperature:

(12) $$a\left(n,T\right)=\frac{69.939n+313.228}{{\left(563.02-T\right)}^{1/2}}$$

We also showed the surface area $a_{-CH2-}$ of the methylene group depends on the temperature and presented the following Equation:

(13) $$a_{-CH2-}(in{Å}^{2})=\frac{69.939}{{\left(563.02-T\right)}^{1/2}}$$

Another expression of the surface area $a_{X}\left(T\right)$ of a polar molecule X was given as a function of temperature:

(14) $$a_{X}\left(T\right)=a_{Xmin.}\times \frac{\left(T_{Max.1}-T\right)\;}{{\left(563.02-T\right)}^{1/2}{\left(T_{Max.\left(X\right)}-T\right)}^{1/2}}$$

where $T_{Max.1}$, $T_{Max.\left(X\right)}$, and $a_{Xmin.}$ are constant characteristics of the polar molecules [35].

The London dispersive surface energy of the different catalysts was therefore obtained using Relation 12 given as a function of temperature:

(15) $$RTln\;V_{n}\left(T\right)=\sqrt{\gamma_{s}^{d}\left(T\right)}\;\left[2Na(T)\;\sqrt{\gamma_{l}^{d}(T)}\right]+A(T)$$

**Figure 1:**
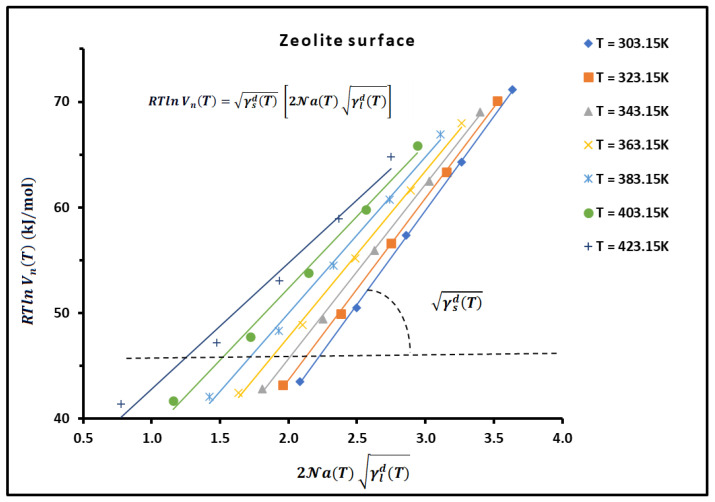
Curves of $RTln\;V_{n}\left(T\right)$ as a function of $\left[2Na(T)\;\sqrt{\gamma_{l}^{d}(T)}\right]$ of n-alkanes (from n-pentane to n-nonane) adsorbed on zeolite material at different temperatures.

Plotting $RTln\;V_{n}\left(T\right)$ as a function of $\left[2Na(T)\;\sqrt{\gamma_{l}^{d}(T)}\right]$ of n-alkanes adsorbed on zeolite material, for example, we obtained a straight line giving a slope equal to $\sqrt{\gamma_{s}^{d}\left(T\right)}$ and therefore the value of $\gamma_{s}^{d}\left(T\right)$ for the different temperatures (Figure 1).

To clarify how exactly the difference in molecular models and temperature dependence affects the values of thermodynamic properties, we determined the values of some surface properties such as the London dispersive surface energy $\gamma_{s}^{d}$ of zeolites at a 1.0% rhodium percentage using the various molecular models compared to our new thermal model. The results of $\gamma_{s}^{d}(T)$ are given in Table 2 at different temperatures for the classic molecular models compared to those obtained by the Hamieh thermal model. Table 2 shows a larger deviation between the previous models and the new thermal model exceeding 400% in certain molecular models.

## 3. Results

### 3.1. Determination of $RTlnV_{n}$ of n-Alkanes Adsorbed on H-β-Zeolite/Rhodium Catalysts

**Figure 2:**
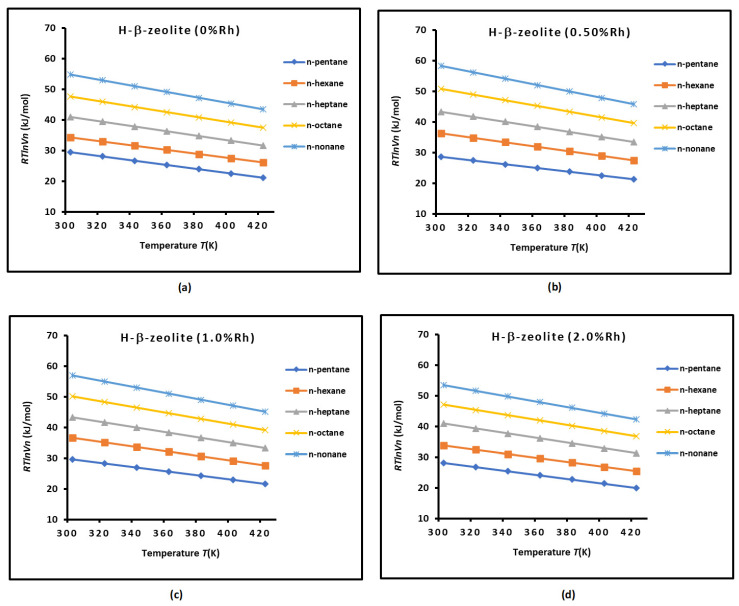
Variations in $RTlnV_{n}$ of n-alkanes of H-β-zeolite as a function of temperature for different rhodium percentages: (**a**) 0% Rh, (**b**) 0. 5% Rh, (**c**) 1.0% Rh, and (**d**) 2.0%Rh.

The chromatographic measurements led to the values of $RTlnV_{n}$ of n-alkanes adsorbed on H-β-zeolite/rhodium at different percentages of rhodium (from 0 to 2%) at different temperatures (in the range of 300 K to 430 K). The experimental results are given in Table S1. The variations in $RTlnV_{n}$ of n-alkanes adsorbed on H-β-zeolite/rhodium are represented in Figure 2 as a function of temperature for various rhodium percentages. The results showed linear variations for the different n-alkanes. A decrease in $RTlnV_{n}(T)$ of n-alkanes adsorbed on zeolites is shown in Figure 2 as a function of temperature. The carbon atom number of n-alkanes affected the values of $RTlnV_{n}(T)$, also depending on the rhodium percentage (Figure 2). This proved an important effect of the rhodium percentage and temperature on the free energy of adsorption of n-alkanes on solid surfaces. The free energy of adsorption decreased when the temperature increased for all n-alkanes and rhodium percentages.

**Figure 3:**
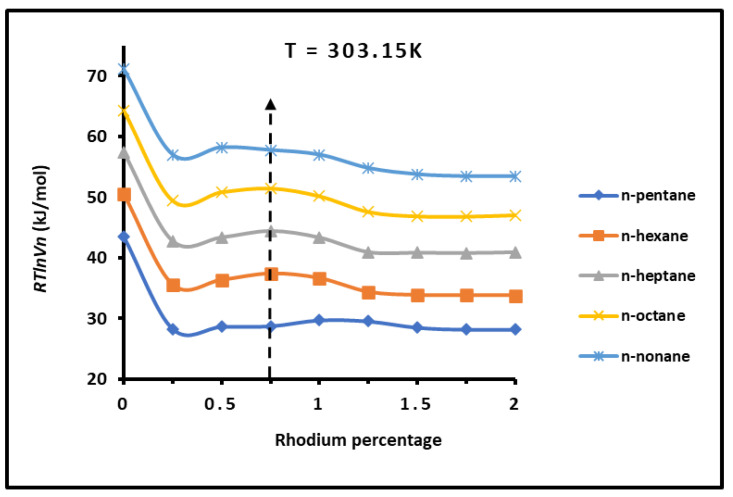
Variations in $RTlnV_{n}$ of n-alkanes adsorbed on H-β-zeolite at 303.15 K as a function of rhodium percentages (%Rh).

The representation of $RTlnV_{n}$ of adsorbed n-alkanes as a function of the rhodium percentage plotted in Figure 3 shows an important decrease in the free energy of adsorption from H-β-zeolite to H-β-zeolite/rhodium. This decrease reached about 20 kJ/mol. The different curves of n-alkanes highlighted a maximum of $RTlnV_{n}$ at a rhodium percentage equal to 0.75% for different temperatures. Figure 3 shows a pallier after 1.0% of rhodium, then showing a stabilization of the free energy for all n-alkanes. There was no effect of the rhodium percentage on the values of $RTlnV_{n}$ and the free energy of adsorption of n-alkanes. This observed phenomenon can be justified by the fact of the saturation of H-β-zeolite by the rhodium for a percentage higher than 1.0%.

### 3.2. London Dispersive Surface Energy of H-β-Zeolite/Rhodium Catalysts

**Figure 4:**
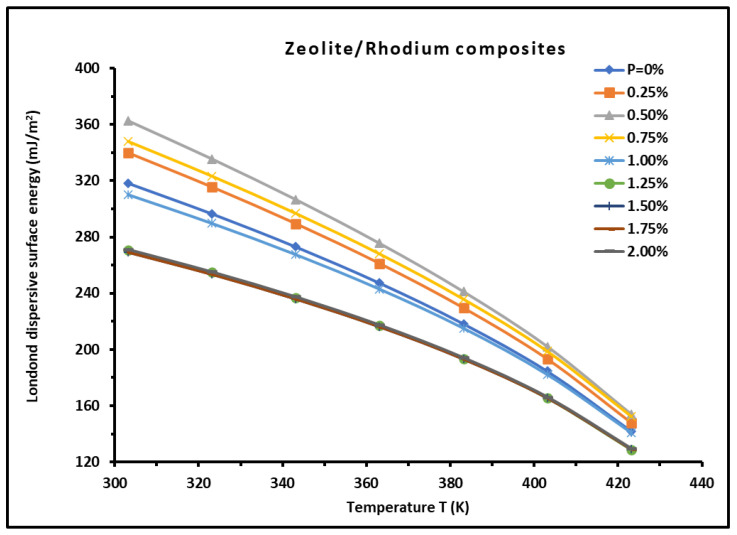
Variations in the London dispersive surface energy $\gamma_{s}^{d}$ of H-β-zeolite/rhodium catalysts as a function of temperature at different rhodium percentages.

The London dispersive surface energy $\gamma_{s}^{d}$ of H-β-zeolite/rhodium catalysts was determined for different percentages of rhodium and various temperatures using Equation (15)

$$RTln\;V_{n}\left(T\right)=\sqrt{\gamma_{s}^{d}\left(T\right)}\;\left[2Na(T)\;\sqrt{\gamma_{l}^{d}(T)}\right]+A(T)$$

and applying the Hamieh thermal model, taking into account the temperature effect on the surface area of n-alkanes a(T) and the London dispersive surface energy of n-alkanes $\gamma_{l}^{d}\left(T\right).$ The representation of $RTln\;V_{n}\left(T\right)$ as a function of $2Na(T)\;\sqrt{\gamma_{l}^{d}(T)}$ of n-alkanes at a fixed temperature led to the values of the slope $\sqrt{\gamma_{s}^{d}\left(T\right)}$ and consequently to the London dispersive surface energy of the different zeolites. The values of $\gamma_{s}^{d}$ of H-β-zeolite/rhodium catalysts are given in Table S2 for different temperatures and rhodium percentages using the straight-line method and Hamieh thermal model. The variations of $\gamma_{s}^{d}$ are plotted in Figure 4 as a function of temperature at different rhodium percentages. A decrease in the values of $\gamma_{s}^{d}(T)$ was observed (Figure 4) versus the temperature and for various rhodium percentages. The variations in $\gamma_{s}^{d}(T)$ were represented by a second-degree equation with an excellent regression coefficient (R^2^ = 0.9993) as follows:

(16) $$\gamma_{s}^{d}\left(T\right)=a\times T^{2}+b\times T+c$$

where the coefficients a, b, and c are constants depending on the solid materials.

Table S2 and Figure 4 allow one to give in Table 3 the different equations for $\gamma_{s}^{d}\left(T\right)$ of the various catalysts. An interesting result can be deduced from Table 3. Indeed, the same value of the coefficient a was obtained for all H-β-zeolite/rhodium catalysts whatever the rhodium percentage, $a=-4.8\times 10^{-3}mJ\times m^{-2}\times K^{-2}$.

It can be concluded from equations in Table 3 that the coefficients a, b, and c are function of the different derivatives of $\gamma_{s}^{d}\left(T\right)$ as follows:

(17) $$\left\{\begin{array}{l} \frac{{d^{2}\gamma }_{s}^{d}}{{dT}^{2}}=2a=-9.6\times 10^{-3}\;\mathrm{mJ}\times m^{-2}\times K^{-2}\\ \frac{{d\gamma }_{s}^{d}}{dT}=b\;\left(in\;mJ\times m^{-2}\times K^{-1}\right)\;\;\;\;\;\;\;\;\;\;\;\;\;\;\;\;\;\;\;\;\;\;\;\;\\ \gamma_{s}^{d}\left(OK\right)=c\;\left(in\;mJ\times m^{-2}\right)\;\;\;\;\;\;\;\;\;\;\;\;\;\;\;\;\;\;\;\;\;\;\;\;\;\;\;\; \end{array}\right.$$

**Figure 5:**
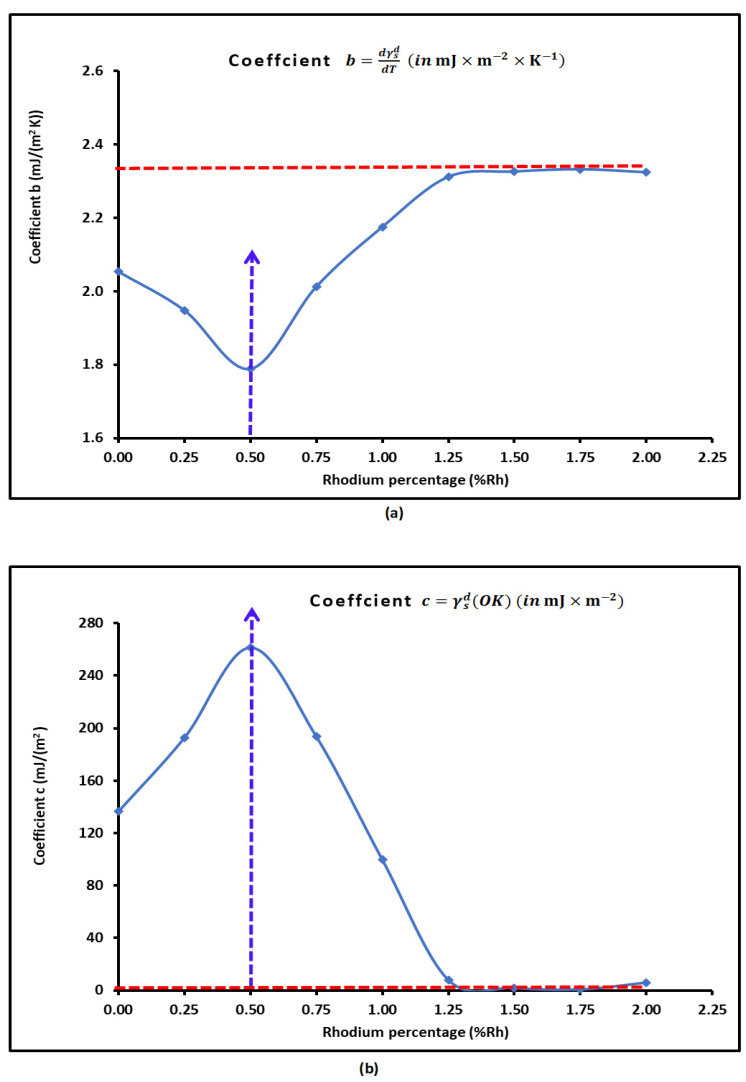
Variations the coefficients $b=\frac{{d\gamma }_{s}^{d}}{dT}$ (**a**) and ${c=\gamma }_{s}^{d}\left(OK\right)$ (**b**) in the general equation of $\gamma_{s}^{d}(T)$ of H-β-zeolite/rhodium catalysts as a function of the rhodium percentage.

The variations in the coefficients b and c are represented in Figure 5 as a function of the rhodium percentage represented by θ. The curves of coefficients b(θ) and c(θ) shown in Figure 5 had parabolic variations for 0≤θ≤1.25%, with a minimum of b(θ) for θ=0.50% and a maximum of c(θ) for the same rhodium percentage, both followed by a pallier.

The results in Figure 5 led to the following expressions of b(θ) and c(θ) in the interval 0≤θ≤1.25%:

(18) $$b\left(\theta \right)=0.712\times \theta^{2}-0.639\times \theta +2.040$$

(19) $$c\left(\theta \right)=-397.48\times \theta^{2}+383.53\times \theta +163.73$$

**Figure 6:**
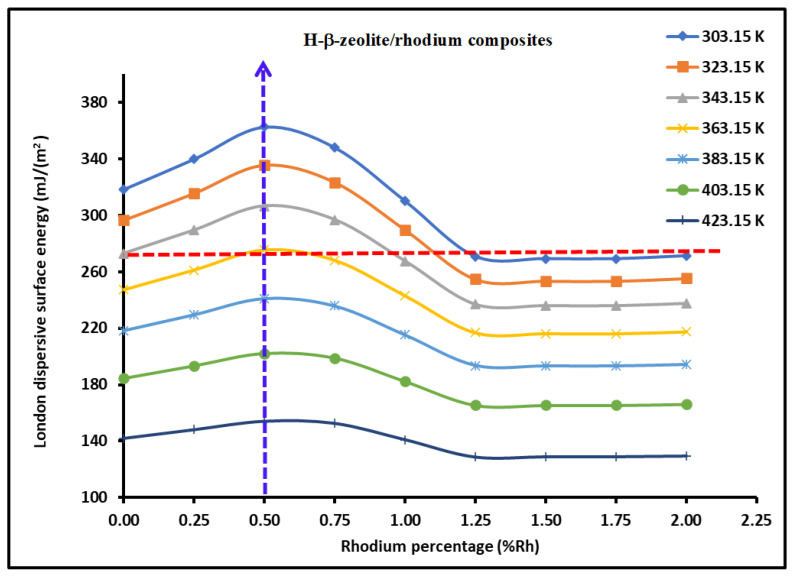
Variations $\gamma_{s}^{d}\left(T,\theta \right)$ of H-β-zeolite/rhodium catalysts as a function of the rhodium percentage θ for the various temperatures.

These equations led to the conclusion that the London dispersive surface energy of H-β-zeolite/rhodium catalysts can be written as a function of two variables (T, θ) as follows:

(20) $$\gamma_{s}^{d}\left(T,\theta \right)=-4.8\times 10^{-3}\times T^{2}+b(\theta )\times T+c(\theta )$$

where b(θ) and c(θ) are given by Equations (18) and (19) in the interval 0≤θ≤1.25%. We observed that $\gamma_{s}^{d}\left(T,\theta \right)$ is constant, independent from the rhodium percentage θ for θ>1.25%. These results were perfectly confirmed by the variations in $\gamma_{s}^{d}\left(T,\theta \right)$ plotted in Figure 6 as a function of θ for different values of temperature. A maximum of $\gamma_{s}^{d}$ was also confirmed at θ=0.50%.

**Figure 7:**
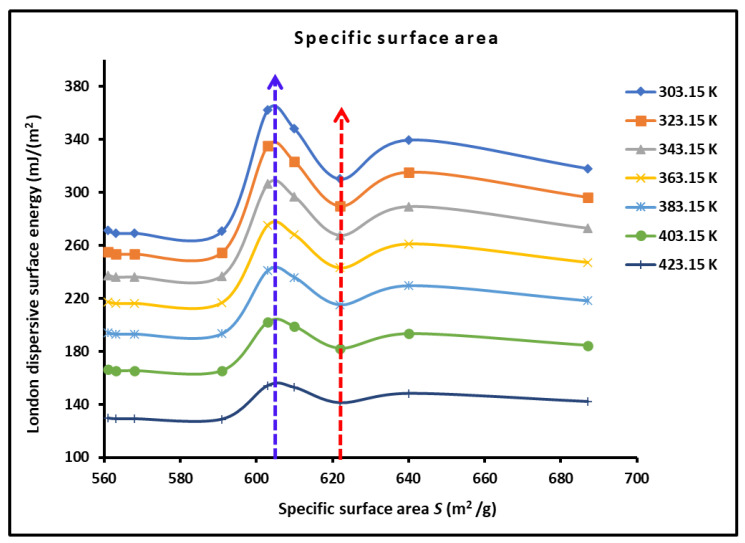
Variations in $\gamma_{s}^{d}\left(T\right)\;(mJ/m^{2})$ of H-β-zeolite/rhodium catalysts as a function of the specific surface area $S_{BET}\;(m^{2}/g)$ for different temperatures.

We plotted the variations $\gamma_{s}^{d}\left(T\right)$ of H-β-zeolite/rhodium catalysts in Figure 7 as a function of the specific surface area $S_{BET}$ using the values in Table 1. The maximum of $\gamma_{s}^{d}$ was obtained for $S_{BET}=603\;m^{2}/g$, corresponding to a rhodium percentage equal to θ=0.50%. A minimum of $\gamma_{s}^{d}$ was obtained at $S_{BET}=622\;m^{2}/g$, corresponding to θ=1.0%, whereas the London dispersive surface energy became constant for θ≥1.25%, certainly due to the effect of the rhodium percentage of the surface energy of the catalyst.

### 3.3. Dispersive Adhesion Work of n-Alkanes on H-β-Zeolite/Rhodium Catalysts

**Figure 8:**
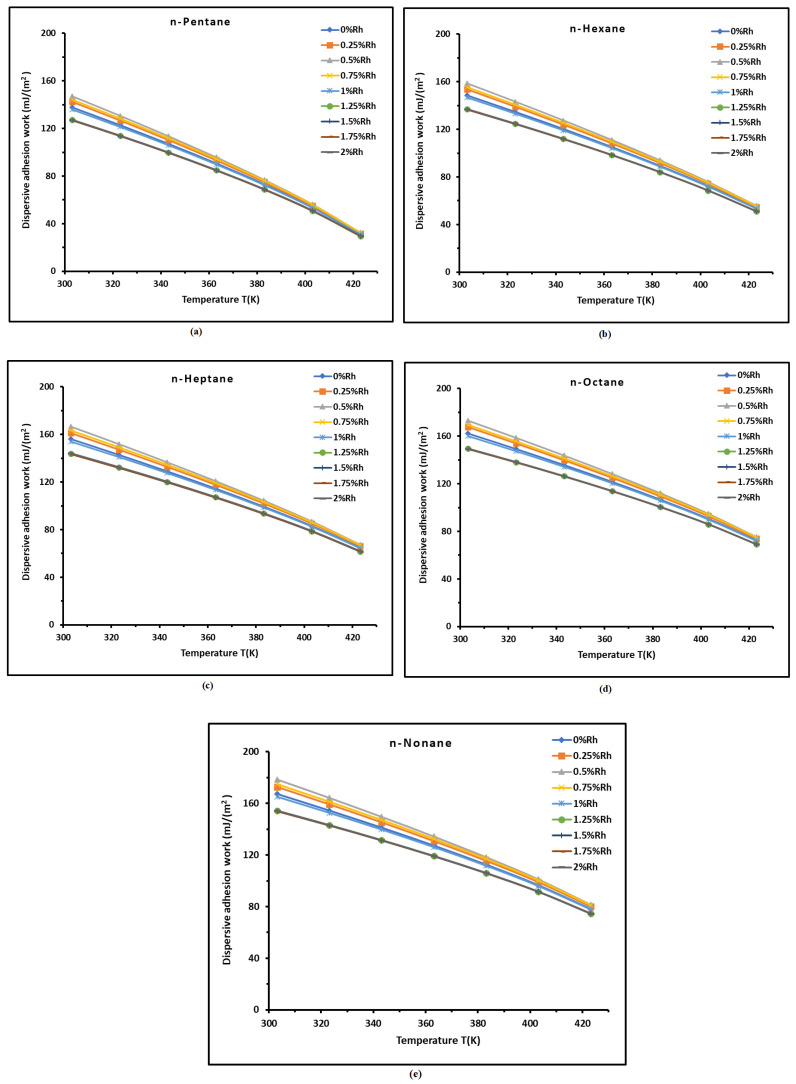
Variations in $W_{a}^{d}(T)$ of the different n-alkanes adsorbed on H-β-zeolite/rhodium catalysts plotted as a function of temperature at different rhodium percentages: (**a**) n-pentane, (**b**) n-hexane, (**c**) n-heptane, (**d**) n-octane, and (**e**) n-nonane.

By applying Equation (8)

$$W_{a}^{d}(T)=2\;\sqrt{\gamma_{l}^{d}\gamma_{s}^{d}}$$

it was possible to calculate the dispersive work of adhesion. The various dispersive adhesion works $W_{a}^{d}$ of the different n-alkanes on H-β-zeolite/rhodium catalysts were determined as a function of temperature. The results are given in Table S3. The corresponding curves of the variations in $W_{a}^{d}(T)$ of the different n-alkanes are plotted in Figure 8. The results showed that the dispersive adhesion work increases when the carbon atom number of n-alkanes increases, while a decrease in $W_{a}^{d}$ was observed when the temperature increases (Figure 8). This result is a direct consequence of the decrease in the London dispersive surface energies of the solid surface and the organic solvents when the temperature decreases.

Furthermore, the curves plotted in Figure 8 showed linear variations in $W_{a}^{d}(T)$ versus the temperature. The equations for $W_{a}^{d}(T)$ of the different n-alkanes adsorbed on H-β-zeolite/rhodium catalysts are given in Table 4 as a function of temperature at different rhodium percentages. The excellent linearity of $W_{a}^{d}(T)$ showed that the dispersive adhesion work can be represented by the following:

(21) $$W_{a}^{d}\left(T\right)=H_{S}^{d}-TS_{S}^{d}$$

where $H_{S}^{d}$ and $S_{S}^{d}$ are, respectively, the dispersive surface enthalpy and entropy of adhesion given in Table 4 for the different n-alkanes adsorbed on H-β-zeolite/rhodium catalysts.

**Figure 9:**
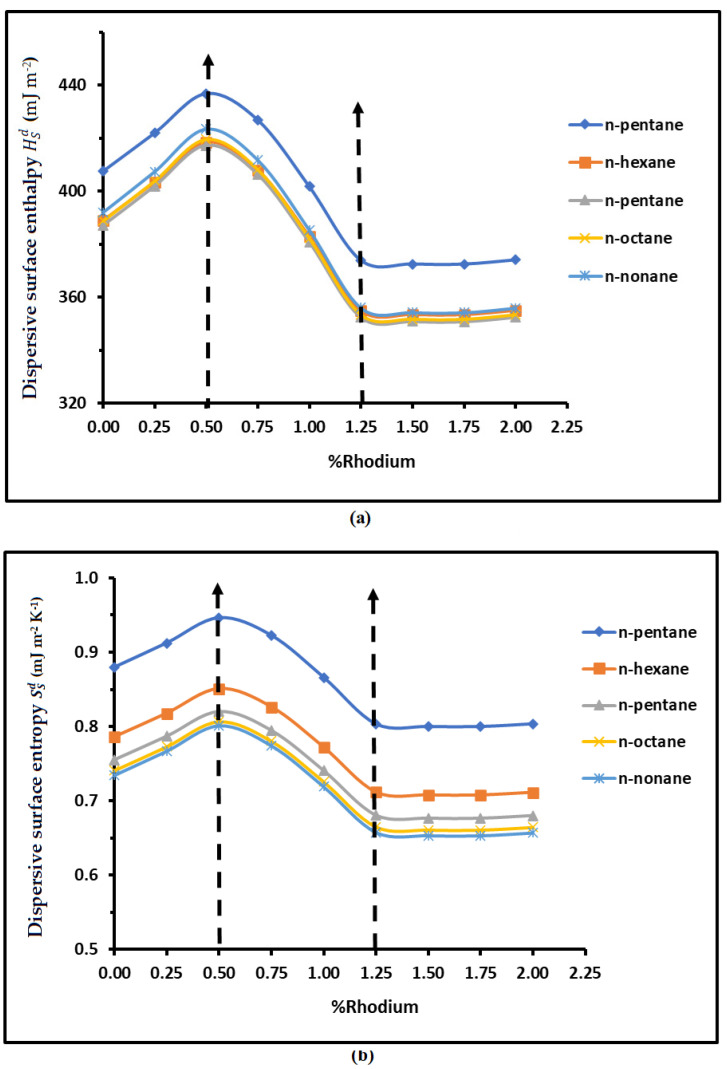
Variations in the dispersive surface enthalpy $H_{S}^{d}$ (mJ m^−2^) (**a**) and $S_{S}^{d}$ (mJ m^−2^ K^−1^) (**b**) of n-alkanes adsorbed on H-β-zeolite/rhodium catalysts as a function of the rhodium percentage.

The results in Table 4 led to the variations in the dispersive surface enthalpy $H_{S}^{d}$ (mJ m^−2^) and entropy $S_{S}^{d}$ (mJ m^−2^ K^−1^) of n-alkanes adsorbed on H-β-zeolite/rhodium catalysts as a function of the rhodium percentage, plotted in Figure 9.

The dispersive surface parameters $H_{S}^{d}$ and $S_{S}^{d}$ given in Figure 9 strongly depended on the rhodium percentage θ. Two zones were distinguished for the above surface variables. The first one was characterized by parabolic variations in the interval [θ=0%; θ=1.25%], with a maximum obtained at θ=0.50%, while a constant pallier for the curves of $H_{S}^{d}$ and $S_{S}^{d}$ was observed after θ=1.25%. The results in Table 5 and Figure 9 show the parabolic variations allowed, giving the different second-degree equations of $H_{S}^{d}(\theta )$ and $S_{S}^{d}(\theta )$.

From Table 5, we can deduce the general equations $H_{S}^{d}(\theta )$ and $S_{S}^{d}(\theta )$ of n-alkanes adsorbed on H-β-zeolite/rhodium catalysts:

(22) $$H_{S}^{d}(\theta )=a\;\theta^{2}+b\;\theta +c$$

(23) $$S_{S}^{d}(\theta )=0.235\;\theta^{2}-0.232\;\theta +d$$

where a, b, c, and d are coefficients depending on the carbon atom number n present in the general formula $C_{n}H_{2n+2}$ of n-alkanes.

The coefficients a, b, c, and d obtained using Table 5 and Equations (22) and (23) are given in Table 6 as a function of the carbon atom number n.

The results in Table 6 led to the conclusion that the dispersive surface enthalpy and entropy of n-alkanes are a function of the rhodium percentage and the carbon atom number of n-alkanes and can therefore be written as $H_{S}^{d}(\theta ,n)$ and $S_{S}^{d}(\theta ,n)$. The dispersive adhesion work of n-alkanes adsorbed on H-β-zeolite/rhodium catalysts can be given by Equation (24):

(24) $$W_{a}^{d}\left(T,\theta ,n\right)=H_{S}^{d}(\theta ,n)-TS_{S}^{d}(\theta ,n)$$

The dispersive adhesion work is then a function of the temperature, rhodium percentage, and the carbon atom number. This new and original result proved for the first time that the adhesion work is a function of three surface variables T, θ, and n.

## 4. Conclusions

The 2D inverse gas chromatography technique at infinite dilution was used to determine the dispersive surface properties of H-β-zeolite/rhodium catalysts at different rhodium percentages by varying the temperature. The chromatographic measurements led to the values of the net retention volume of adsorption of n-alkanes on the solid surfaces and therefore to the dispersive free energy of adsorption. The use of our new thermal model, taking into account the temperature effect on the surface area of n-alkanes, allowed one to determine accurate values of the London dispersive surface energy $\gamma_{s}^{d}\left(T\right)$ as a function of temperature. The results showed parabolic variations in $\gamma_{s}^{d}\left(T,\theta \right)$ as a function of the temperature T and the rhodium coefficient θ. A similar variation in $\gamma_{s}^{d}$ as a function of the specific surface area of solid catalysts was also observed. The dispersive adhesion work, surface enthalpy, and surface entropy of n-alkanes adsorbed on H-β-zeolite/rhodium catalysts were determined. The results showed linear variations in the adhesion work $W_{a}^{d}\left(T\right)$ as a function of temperature for a fixed rhodium percentage. However, parabolic variations of the adhesion work $W_{a}^{d}\left(\theta ,n\right)$ were observed as a function of the rhodium percentage and the carbon atom number of n-alkanes. When the rhodium percentage and the carbon atom number varied, it was showed that the dispersive adhesion work $W_{a}^{d}\left(T,\;\theta ,n\right)$ was a function of three variables: temperature, rhodium percentage, and the carbon atom number of n-alkanes.

## Figures and Tables

**Table 1:** Values of the specific surface area $S_{BET}$ (m^2^/g) and microporous volume $V_{m}$ (cm^3^/g) of the various catalysts [32].

%Rh	S_BET_ (m^2^/g)	V_m_ (cm^3^/g)
0	687	0.198
0.25	640	0.185
0.5	603	0.175
0.75	610	0.177
1	622	0.182
1.25	591	0.172
1.5	568	0.165
1.75	563	0.164
2	561	0.163

**Table 2:** Values of the London dispersive surface energy $\gamma_{s}^{d}$ of zeolites at 1.0% rhodium percentage at various temperatures using the various molecular models compared to our new thermal model, and the deviation percentage between the classic molecular models and the Hamieh thermal model.

Temperature (K)	303.15	323.15	343.15	363.15	383.15	403.15	423.15
Van der Waals	287.70	285.49	283.27	281.06	278.85	276.63	274.42
Kiselev	269.10	266.15	263.19	260.23	257.27	254.31	251.36
Cylindrical	224.45	222.38	220.30	218.23	216.15	214.07	212.00
Geometric	148.60	147.17	145.74	144.31	142.88	141.45	140.02
Redlich–Kwong	470.50	466.91	463.32	459.72	456.13	452.53	448.94
Spherical	961.77	953.42	945.07	936.71	928.36	920.00	911.65
Gray	295.22	291.87	288.52	285.17	281.82	278.47	275.12
Hamieh	310.18	289.78	267.62	243.05	215.27	182.33	141.06
Deviation percentage							
T (K)	303.15	323.15	343.15	363.15	383.15	403.15	423.15
Van der Waals	−7.25	−1.48	5.85	15.64	29.53	51.72	94.54
Kiselev	−13.24	−8.16	−1.65	7.07	19.51	39.48	78.19
Cylindrical	−27.64	−23.26	−17.68	−10.21	0.41	17.41	50.29
Geometric	−52.09	−49.21	−45.54	−40.62	−33.62	−22.42	−0.74
Redlich–Kwong	51.69	61.12	73.13	89.15	111.89	148.19	218.25
Spherical	210.07	229.01	253.14	285.40	331.26	404.58	546.27
Gray	−4.82	0.72	7.81	17.33	30.92	52.73	95.03

**Table 3:** Values of $\gamma_{s}^{d}(T)$ of different catalysts as a function of temperature, rhodium percentage, and molecular model using the increment method.

**%Rh**	$\mathrm{Equations}\;\gamma_{s}^{d}\left(T\right)$ of Catalysts	R^2^
0.00%	$\gamma_{s}^{d}\left(T\right)=-4.8\times 10^{-3}T^{2}+2.053\times T+136.62$	0.9993
0.25%	$\gamma_{s}^{d}\left(T\right)=-4.8\times 10^{-3}T^{2}+1.948\times T+192.94$	0.9994
0.50%	$\gamma_{s}^{d}\left(T\right)=-4.8\times 10^{-3}T^{2}+1.789\times T+261.53$	0.9995
0.75%	$\gamma_{s}^{d}\left(T\right)=-4.8\times 10^{-3}T^{2}+2.013\times T+193.7$	0.9994
1.00%	$\gamma_{s}^{d}\left(T\right)=-4.8\times 10^{-3}T^{2}+2.175\times T+99.66$	0.9993
1.25%	$\gamma_{s}^{d}\left(T\right)=-4.8\times 10^{-3}T^{2}+2.311\times T+7.848$	0.999
1.50%	$\gamma_{s}^{d}\left(T\right)=-4.8\times 10^{-3}T^{2}+2.326\times T+1.712$	0.999
1.75%	$\gamma_{s}^{d}\left(T\right)=-4.8\times 10^{-3}T^{2}+2.332\times T+0.584$	0.999
2.00%	$\gamma_{s}^{d}\left(T\right)=-4.8\times 10^{-3}T^{2}+2.324\times T+5.795$	0.999

**Table 4:** Equations of $W_{a}^{d}(T)$ (mJ m^−2^), $H_{S}^{d}$ (mJ m^−2^), and $S_{S}^{d}$ (mJ m^−2^ K^−1^) of the different n-alkanes adsorbed on H-β-zeolite/rhodium catalysts as a function of temperature at different rhodium percentages, with the corresponding values of the linear regression coefficients.

**n-pentane**				
**%Rhodium**	$W_{a}^{d}(T)$ **(mJ m^−2^)**	$S_{S}^{d}$ **(mJ m^−2^ K^−1^)**	$H_{S}^{d}$ **(mJ m^−2^)**	**R^2^**
0	$W_{a}^{d}(T)$ = −0.8797 T + 407.51	0.8797	407.51	0.995
0.25	$W_{a}^{d}\left(T\right)\;$= −0.9123 T + 421.94	0.9123	421.94	0.9955
0.5	$W_{a}^{d}(T)$ = −0.9463 T + 436.84	0.9463	436.84	0.996
0.75	$W_{a}^{d}(T)$ = −0.9226 T + 426.85	0.9226	426.85	0.9955
1	$W_{a}^{d}(T)$ = −0.866 T + 401.7	0.866	401.7	0.9946
1.25	$W_{a}^{d}(T)$ = −0.8032 T + 373.84	0.8032	373.84	0.9935
1.5	$W_{a}^{d}(T)$ = −0.7998 T + 372.46	0.7998	372.46	0.9933
1.75	$W_{a}^{d}(T)$ = −0.7997 T + 372.42	0.7997	372.42	0.9933
2	$W_{a}^{d}(T)$ = −0.8034 T + 374.02	0.8034	374.02	0.9934
**n-hexane**				
**%Rhodium**	$W_{a}^{d}(T)$ **(mJ m^−2^)**	$S_{S}^{d}$ **(mJ m^−2^ K^−1^)**	$H_{S}^{d}$ **(mJ m^−2^)**	**R^2^**
0	$W_{a}^{d}(T)$ = −0.786 T + 388.86	0.786	388.86	0.9973
0.25	$W_{a}^{d}(T)$ = −0.8177 T + 403.34	0.8177	403.34	0.9976
0.5	$W_{a}^{d}(T)$ = −0.8509 T + 418.39	0.8509	418.39	0.998
0.75	$W_{a}^{d}(T)$ = −0.8264 T + 407.87	0.8264	407.87	0.9976
1	$W_{a}^{d}(T)$ = −0.772 T + 382.78	0.772	382.78	0.997
1.25	$W_{a}^{d}(T)$ = −0.7115 T + 354.95	0.7115	354.95	0.9961
1.5	$W_{a}^{d}(T)$ = −0.7077 T + 353.42	0.7077	353.42	0.996
1.75	$W_{a}^{d}(T)$ = −0.7076 T + 353.38	0.7076	353.38	0.9961
2	$W_{a}^{d}(T)$ = −0.7112 T + 355.01	0.7112	355.01	0.9961
**n-heptane**				
**%Rhodium**	$W_{a}^{d}(T)$ **(mJ m^−2^)**	$S_{S}^{d}$ **(mJ m^−2^ K^−1^)**	$H_{S}^{d}$ **(mJ m^−2^)**	**R^2^**
0	$W_{a}^{d}(T)$ = −0.7554 T + 387.11	0.7554	387.11	0.9971
0.25	$W_{a}^{d}(T)$ = −0.7872 T + 401.88	0.7872	401.88	0.9975
0.5	$W_{a}^{d}(T)$ = −0.8206 T + 417.27	0.8206	417.27	0.9978
0.75	$W_{a}^{d}(T)$ = −0.7952 T + 406.3	0.7952	406.3	0.9974
1	$W_{a}^{d}(T)$ = −0.7408 T + 380.79	0.7408	380.79	0.9968
1.25	$W_{a}^{d}(T)$ = −0.6804 T + 352.47	0.6804	352.47	0.9959
1.5	$W_{a}^{d}(T)$ = −0.6764 T + 350.84	0.6764	350.84	0.9958
1.75	$W_{a}^{d}(T)$ = −0.6763 T + 350.8	0.6763	350.8	0.9958
2	$W_{a}^{d}(T)$ = −0.68 T + 352.47	0.68	352.47	0.9959
**n-octane**				
**%Rhodium**	$W_{a}^{d}(T)$ **(mJ m^−2^)**	$S_{S}^{d}$ **(mJ m^−2^ K^−1^)**	$H_{S}^{d}$ **(mJ m^−2^)**	**R^2^**
0	$W_{a}^{d}(T)$ = −0.7408 T + 388.78	0.7408	388.78	0.9967
0.25	$W_{a}^{d}(T)$ = −0.7729 T + 403.85	0.7729	403.85	0.9971
0.5	$W_{a}^{d}(T)$ = −0.8067 T + 419.57	0.8067	419.57	0.9975
0.75	$W_{a}^{d}(T)$ = −0.7805 T + 408.24	0.7805	408.24	0.997
1	$W_{a}^{d}(T)$ = −0.7258 T + 382.27	0.7258	382.27	0.9964
1.25	$W_{a}^{d}(T)$ = −0.665 T + 353.43	0.665	353.43	0.9954
1.5	$W_{a}^{d}(T)$ = −0.6608 T + 351.72	0.6608	351.72	0.9952
1.75	$W_{a}^{d}(T)$ = −0.6607 T + 351.68	0.6607	351.68	0.9953
2	$W_{a}^{d}(T)$ = −0.6644 T + 353.39	0.6644	353.39	0.9953
**n-nonane**				
**%Rhodium**	$W_{a}^{d}(T)$ **(mJ m^−2^)**	$S_{S}^{d}$ **(mJ m^−2^ K^−1^)**	$H_{S}^{d}$ **(mJ m^−2^)**	**R^2^**
0	$W_{a}^{d}(T)$ = −0.7343 T + 392.02	0.7343	392.02	0.9962
0.25	$W_{a}^{d}(T)$ = −0.7667 T + 407.38	0.7667	407.38	0.9966
0.5	$W_{a}^{d}(T)$ = −0.801 T + 423.42	0.801	423.42	0.9971
0.75	$W_{a}^{d}(T)$ = −0.7742 T + 411.77	0.7742	411.77	0.9966
1	$W_{a}^{d}(T)$ = −0.719 T + 385.33	0.719	385.33	0.9959
1.25	$W_{a}^{d}(T)$ = −0.6576 T + 355.97	0.6576	355.97	0.9948
1.5	$W_{a}^{d}(T)$ = −0.6532 T + 354.2	0.6532	354.2	0.9947
1.75	$W_{a}^{d}(T)$ = −0.6531 T + 354.15	0.6531	354.15	0.9947
2	$W_{a}^{d}(T)$ = −0.6569 T + 355.9	0.6569	355.9	0.9948

**Table 5:** Equations of the dispersive surface enthalpy $H_{S}^{d}(\theta )$ (mJ m^−2^) and entropy $S_{S}^{d}(\theta )$ (mJ m^−2^ K^−1^) of n-alkanes adsorbed on H-β-zeolite/rhodium catalysts as a function of the rhodium percentage, with the linear regression coefficient R^2^ and the general equation.

**n-Alkanes**	$\mathrm{Dispersive}\;\mathrm{Surface}\;\mathrm{Enthalpy}\;H_{S}^{d}$	R^2^	$\mathrm{Dispersive}\;\mathrm{Surface}\;\mathrm{Entropy}\;S_{S}^{d}$	R^2^
n-pentane	$H_{S}^{d}$ = −106.2 θ^2^ + 105.4 θ + 406.4	0.9777	$S_{S}^{d}$ = 0.24 θ^2^ − 0.24 θ − 0.877	0.977
n-hexane	$H_{S}^{d}$ = −106.3 θ^2^ + 105.3 θ + 387.82	0.9771	$S_{S}^{d}$ = 0.23 θ^2^ − 0.23 θ − 0.784	0.9762
n-pentane	$H_{S}^{d}$= −108.3 θ^2^ + 107.1 θ + 386.08	0.9768	$S_{S}^{d}$ = 0.23 θ^2^ − 0.23 θ − 0.754	0.9756
n-octane	$H_{S}^{d}$= −110.4 θ^2^ + 109.1 θ + 387.75	0.9766	$S_{S}^{d}$ = 0.23 θ^2^ − 0.23 θ − 0.739	0.9752
n-nonane	$H_{S}^{d}$= −112.4 θ^2^ + 111.1 θ + 390.99	0.9765	$S_{S}^{d}$ = 0.24 θ^2^ − 0.23 θ − 0.732	0.9749

**Table 6:** Equations of the coefficients a(n), b(n), c(n), and d(n) as a function of the carbon atom number n with the linear regression coefficient R^2^.

a(n)= −0.282 n^2^ + 2.297 n− 110.41	R^2^ = 0.9866
b(n)= 0.313 n^2^ − 2.872 n+ 111.73	R^2^ = 0.9827
c(n)= 3.361 n^2^ − 50.139 n+ 571.38	R^2^ = 0.9095
d(n)= −0.014 n^2^ + 0.223 n− 1.648	R^2^ = 0.9770

## Data Availability

There are no additional data.

## Supplementary Materials

The following supporting information can be downloaded at: https://www.mdpi.com/article/10.3390/ma18010081/s1, Table S1. Values of RTlnVn (kJ/mol) of n-alkanes adsorbed on H-β-zeolite/rhodium composites as a function of the temperature for different rhodium percentages (%Rh = 0; 0.25; 0.50; 0.75; 1.00; 1.25; 1.50; 1.75; 2.00). Table S2. Values of the London dispersive surface energy $\gamma_{s}^{d}$ $\left(mJ/m^{2}\right)$ of H-β-zeolite/rhodium composites as a function of temperature and rhodium percentage using the straight-line method and Hamieh thermal model. Table S3. Variation in dispersive adhesion work $W_{a}^{d}(mJ/m^{2})of\;n-alkanes$ on H-β-zeolite/rhodium composites as a function of temperature and rhodium percentages.

## List of Abbreviations

a (${Å}^{2}):$	the surface area of an adsorbed molecule
A×(T):	constant depending on temperature
*AN’*:	the corrected acceptor number of electrons of a probe
$a_{X}\left(T\right)$ (${Å}^{2}):$	the surface area of a polar molecule X
$C_{n}H_{2(n+1)}$	the general formula of n-alkane
$D_{c}$ (cm^3^/min):	the corrected flow rate
$D_{m}$ (cm^3^/min):	the measured flow rate
*DN’*	the donor number of electrons of a probe
$H_{S}^{d}$ (mJ m^−2^):	the dispersive surface enthalpy of adhesion
IGC	inverse gas chromatography
j	the James–Martin coefficient
*m* (g):	the mass of a solid
N	the Avogadro’s number
$P_{0}$ (Pa):	the pressure of gas
*R*	the perfect gas constant
*R^2^*	the linear regression coefficient
*s* (m^2^/g):	the specific surface area
$S_{BET}$ ($m^{2}/g$):	the specific surface area of a solid material
$S_{S}^{d}$ (mJ m^−2^ K^−1^)	the entropy of adhesion
$t_{0}$ (min):	the zero-retention reference time
$t_{R}$ (min)	the retention time of an adsorbed solvent
*T* (K):	the absolute temperature
$T_{a}$ (K)	the room temperature
$T_{B.P.}$ (K):	the boiling point of a solvent
$T_{Max.1}$, $T_{Max.\left(X\right)}$, and $a_{Xmin.}$	are constant characteristics of the polar molecules
$V_{m}$ (cm^3^/g):	microporous volume of catalyst
$V_{n}$ (cm^3^):	the net retention volume considered
$W_{a}^{d}(T)$ (mJ/m^2^)	the dispersive work of adhesion
$x_{j}$	surface chromatographic parameter
$\alpha_{0}$:	the deformation polarizability of a molecule
$\alpha_{j}$ and $\beta_{j}$	constants relative to the adsorption of n-alkanes on solid surfaces
$\gamma_{l}^{d}$ (mJ/m^2^):	the dispersive component of the liquid solvent
$\gamma_{s}^{d}$ (mJ/m^2^):	the dispersive component of the solid
$∆G_{a}^{0}$ (kJ/mol):	the standard free energy of adsorption
$\left(-∆G_{a}^{d}\right)$ (kJ/mol):	the free dispersive energy of adsorption
$∆G_{-CH2-}^{0}$ (kJ/mol):	the free energy of the methylene group
$\left(-∆H_{a}^{p}\right)$ (kJ/mol):	the polar enthalpy of adsorption
$\left(-∆S_{a}^{p}\right)$ (J mol^−1^ K^−1^):	the polar entropy of adsorption
ΔP (Pa)*:*	the pressure variation
η	the gas viscosity (cp)
θ	the rhodium percentage
$\pi_{0}$ (N/m):	the two-dimensional pressure or spreading pressure
$\chi_{T}$:	the topological index of an organic molecule
