# Supplementary material for: Application of a New Thermal Model for the Determination of London Dispersive Properties of H-β-Zeolite/Rhodium Catalysts Using New 2D Chromatographic Models
Source: Materials (Basel). 2024 Dec 28;18(1):81. doi: 10.3390/ma18010081 (PMC11722188; doi:10.3390/ma18010081)
Supplement: Supplementary file 1 [file materials-18-00081-s001.zip › materials-3348782-supplementary.pdf]

## Supplementary Materials

# Application of a New Thermal Model for the Determination of London Dispersive Properties of H-β-Zeolite/Rhodium Catalysts Using New 2D Chromatographic Models

**Tayssir Hamieh[1,2,3]*

[1]Faculty of Science and Engineering, Maastricht University, P.O. Box 616, 6200 MD Maastricht, Netherlands.
[2]Institut de Science des Matériaux de Mulhouse, Université de Haute-Alsace, CNRS, IS2M UMR 7361, F-68100 Mulhouse, France
[3]Laboratory of Materials, Catalysis, Environment and Analytical Methods (MCEMA), Faculty of Sciences, Lebanese University, Hadath, Lebanon.

*Correspondence: E-mail: t.hamieh@maastrichtuniversity.nl

**Table S1.** Values of $RTlnVn$ (kJ/mol) of n-alkanes adsorbed on H-β-zeolite/rhodium composites as a function of the temperature for different rhodium percentages (%Rh = 0; 0.25; 0.50; 0.75; 1.00; 1.25; 1.50; 1.75; 2.00).

| 0% | | | | | | | |
|---|---|---|---|---|---|---|---|
| n-alkanes | 303.15 K | 323.15 K | 343.15 K | 363.15 K | 383.15 K | 403.15 K | 423.15 K |
| n-pentane | 43.542 | 43.180 | 42.818 | 42.456 | 42.094 | 41.732 | 41.370 |
| n-hexane | 50.513 | 49.965 | 49.417 | 48.869 | 48.321 | 47.773 | 47.225 |
| n-heptane | 57.341 | 56.631 | 55.921 | 55.211 | 54.501 | 53.791 | 53.081 |
| n-octane | 64.264 | 63.376 | 62.488 | 61.600 | 60.712 | 59.824 | 58.936 |
| n-nonane | 71.165 | 70.103 | 69.041 | 67.979 | 66.917 | 65.855 | 64.793 |
| 0.25% | | | | | | | |
| n-alkanes | 303.15 K | 323.15 K | 343.15 K | 363.15 K | 383.15 K | 403.15 K | 423.15 K |
| n-pentane | 28.289 | 26.975 | 25.661 | 24.347 | 23.033 | 21.719 | 20.405 |
| n-hexane | 35.565 | 34.035 | 32.505 | 30.975 | 29.445 | 27.915 | 26.385 |
| n-heptane | 42.764 | 41.044 | 39.324 | 37.604 | 35.884 | 34.164 | 32.444 |
| n-octane | 49.430 | 47.560 | 45.690 | 43.820 | 41.950 | 40.080 | 38.210 |
| n-nonane | 57.007 | 54.905 | 52.803 | 50.701 | 48.599 | 46.497 | 44.395 |
| 0.50% | | | | | | | |
| n-alkanes | 303.15 K | 323.15 K | 343.15 K | 363.15 K | 383.15 K | 403.15 K | 423.15 K |
| n-pentane | 28.647 | 27.425 | 26.203 | 24.981 | 23.759 | 22.537 | 21.315 |
| n-hexane | 36.348 | 34.878 | 33.408 | 31.938 | 30.468 | 28.998 | 27.528 |
| n-heptane | 43.373 | 41.729 | 40.085 | 38.441 | 36.797 | 35.153 | 33.509 |
| n-octane | 50.840 | 48.972 | 47.104 | 45.236 | 43.368 | 41.500 | 39.632 |

| | | | | | | | |
|---|---|---|---|---|---|---|---|
| n-nonane | 58.219 | 56.141 | 54.063 | 51.985 | 49.907 | 47.829 | 45.751 |
| | | | 0.75% | | | | |
| n-alkanes | 303.15 K | 323.15 K | 343.15 K | 363.15 K | 383.15 K | 403.15 K | 423.15 K |
| n-pentane | 28.738 | 27.558 | 26.378 | 25.198 | 24.018 | 22.838 | 21.658 |
| n-hexane | 37.402 | 35.888 | 34.374 | 32.860 | 31.346 | 29.832 | 28.318 |
| n-heptane | 44.426 | 42.746 | 41.066 | 39.386 | 37.706 | 36.026 | 34.346 |
| n-octane | 51.459 | 49.605 | 47.751 | 45.897 | 44.043 | 42.189 | 40.335 |
| n-nonane | 57.764 | 55.784 | 53.804 | 51.824 | 49.844 | 47.864 | 45.884 |
| | | | 1.00% | | | | |
| n-alkanes | 303.15 K | 323.15 K | 343.15 K | 363.15 K | 383.15 K | 403.15 K | 423.15 K |
| n-pentane | 29.710 | 28.372 | 27.034 | 25.696 | 24.358 | 23.020 | 21.682 |
| n-hexane | 36.623 | 35.121 | 33.619 | 32.117 | 30.615 | 29.113 | 27.611 |
| n-heptane | 43.334 | 41.670 | 40.006 | 38.342 | 36.678 | 35.014 | 33.350 |
| n-octane | 50.170 | 48.342 | 46.514 | 44.686 | 42.858 | 41.030 | 39.202 |
| n-nonane | 56.997 | 55.007 | 53.017 | 51.027 | 49.037 | 47.047 | 45.057 |
| | | | 1.25% | | | | |
| n-alkanes | 303.15 K | 323.15 K | 343.15 K | 363.15 K | 383.15 K | 403.15 K | 423.15 K |
| n-pentane | 29.525 | 28.119 | 26.713 | 25.307 | 23.901 | 22.495 | 21.089 |
| n-hexane | 34.355 | 32.983 | 31.611 | 30.239 | 28.867 | 27.495 | 26.123 |
| n-heptane | 40.946 | 39.402 | 37.858 | 36.314 | 34.770 | 33.226 | 31.682 |
| n-octane | 47.575 | 45.885 | 44.195 | 42.505 | 40.815 | 39.125 | 37.435 |
| n-nonane | 54.773 | 52.881 | 50.989 | 49.097 | 47.205 | 45.313 | 43.421 |
| | | | 1.50% | | | | |
| n-alkanes | 303.15 K | 323.15 K | 343.15 K | 363.15 K | 383.15 K | 403.15 K | 423.15 K |
| n-pentane | 28.494 | 27.126 | 25.758 | 24.390 | 23.022 | 21.654 | 20.286 |
| n-hexane | 33.838 | 32.466 | 31.094 | 29.722 | 28.350 | 26.978 | 25.606 |
| n-heptane | 40.846 | 39.258 | 37.670 | 36.082 | 34.494 | 32.906 | 31.318 |
| n-octane | 46.857 | 45.183 | 43.509 | 41.835 | 40.161 | 38.487 | 36.813 |
| n-nonane | 53.742 | 51.890 | 50.038 | 48.186 | 46.334 | 44.482 | 42.630 |
| | | | 1.75% | | | | |
| n-alkanes | 303.15 K | 323.15 K | 343.15 K | 363.15 K | 383.15 K | 403.15 K | 423.15 K |
| n-pentane | 28.159 | 26.809 | 25.459 | 24.109 | 22.759 | 21.409 | 20.059 |
| n-hexane | 33.811 | 32.429 | 31.047 | 29.665 | 28.283 | 26.901 | 25.519 |
| n-heptane | 40.801 | 39.205 | 37.609 | 36.013 | 34.417 | 32.821 | 31.225 |
| n-octane | 46.814 | 45.128 | 43.442 | 41.756 | 40.070 | 38.384 | 36.698 |
| n-nonane | 53.408 | 51.572 | 49.736 | 47.900 | 46.064 | 44.228 | 42.392 |
| | | | 2.00% | | | | |
| n-alkanes | 303.15 K | 323.15 K | 343.15 K | 363.15 K | 383.15 K | 403.15 K | 423.15 K |
| n-pentane | 28.180 | 26.822 | 25.464 | 24.106 | 22.748 | 21.390 | 20.032 |
| n-hexane | 33.779 | 32.393 | 31.007 | 29.621 | 28.235 | 26.849 | 25.463 |
| n-heptane | 40.931 | 39.313 | 37.695 | 36.077 | 34.459 | 32.841 | 31.223 |
| n-octane | 47.046 | 45.332 | 43.618 | 41.904 | 40.190 | 38.476 | 36.762 |
| n-nonane | 53.422 | 51.578 | 49.734 | 47.890 | 46.046 | 44.202 | 42.358 |

**Table S2**. Values of the London dispersive surface energy $\gamma_s^d$ $(\mathrm{mJ}/m^2)$ of H-β-zeolite/rhodium composites as a function of temperature and rhodium percentage using the straight-line method and Hamieh thermal model.

| %Rh | 303.15 K | 323.15 K | 343.15 K | 363.15 K | 383.15 K | 403.15 K | 423.15 K |
|---|---|---|---|---|---|---|---|
| 0 % | 318.23 | 296.49 | 273.04 | 247.24 | 218.30 | 184.55 | 142.04 |
| 0.25% | 339.74 | 315.49 | 289.48 | 261.18 | 229.64 | 193.35 | 148.13 |
| 0.50% | 362.56 | 335.44 | 306.60 | 275.46 | 241.18 | 202.12 | 154.11 |
| 0.75% | 348.08 | 323.39 | 296.91 | 268.08 | 235.93 | 198.84 | 152.65 |
| 1.0 % | 310.18 | 289.78 | 267.62 | 243.05 | 215.27 | 182.33 | 141.06 |
| 1.25% | 270.90 | 254.82 | 236.98 | 216.86 | 193.54 | 165.48 | 128.80 |
| 1.50% | 269.30 | 253.60 | 236.00 | 216.20 | 193.21 | 165.50 | 129.10 |
| 1.75% | 269.26 | 253.51 | 235.99 | 216.18 | 193.18 | 165.43 | 129.05 |
| 2.0 % | 271.36 | 255.36 | 237.59 | 217.50 | 194.24 | 166.20 | 129.55 |

**Table S3.** Variation of dispersive adhesion work $W_a^d$ $(\mathrm{mJ}/m^2)$ of $\mathrm{n}-$alkanes on H-β-zeolite/rhodium composites as a function of temperature and rhodium percentages.

| n-alkanes | %Rhodium | 303.15K | 323.15K | 343.15K | 363.15K | 383.15K | 403.15K | 423.15K |
|---|---|---|---|---|---|---|---|---|
| C5 | 0 | 137.84 | 122.84 | 107.18 | 90.68 | 73.05 | 53.70 | 31.10 |
| C6 | 0 | 148.51 | 134.62 | 120.23 | 105.19 | 89.35 | 72.38 | 53.57 |
| C7 | 0 | 156.11 | 142.76 | 128.95 | 114.54 | 99.36 | 83.06 | 64.78 |
| C8 | 0 | 162.15 | 149.13 | 135.66 | 121.59 | 106.73 | 90.70 | 72.46 |
| C9 | 0 | 167.25 | 154.43 | 141.16 | 127.27 | 112.57 | 96.62 | 78.26 |
| C5 | 0.25 | 142.43 | 126.71 | 110.36 | 93.20 | 74.92 | 54.97 | 31.76 |
| C6 | 0.25 | 153.45 | 138.87 | 123.79 | 108.12 | 91.64 | 74.09 | 54.71 |
| C7 | 0.25 | 161.30 | 147.27 | 132.78 | 117.72 | 101.91 | 85.02 | 66.15 |
| C8 | 0.25 | 167.54 | 153.83 | 139.68 | 124.97 | 109.47 | 92.84 | 74.00 |
| C9 | 0.25 | 172.81 | 159.30 | 145.34 | 130.81 | 115.46 | 98.90 | 79.92 |
| C5 | 0.5 | 147.13 | 130.66 | 113.58 | 95.72 | 76.78 | 56.20 | 32.40 |
| C6 | 0.5 | 158.52 | 143.19 | 127.40 | 111.03 | 93.92 | 75.75 | 55.80 |
| C7 | 0.5 | 166.63 | 151.85 | 136.65 | 120.90 | 104.43 | 86.92 | 67.47 |
| C8 | 0.5 | 173.07 | 158.62 | 143.75 | 128.34 | 112.19 | 94.92 | 75.48 |
| C9 | 0.5 | 178.52 | 164.26 | 149.58 | 134.34 | 118.32 | 101.12 | 81.52 |
| C5 | 0.75 | 144.16 | 128.29 | 111.77 | 94.42 | 75.94 | 55.74 | 32.25 |
| C6 | 0.75 | 155.32 | 140.59 | 125.37 | 109.53 | 92.89 | 75.13 | 55.53 |
| C7 | 0.75 | 163.27 | 149.10 | 134.47 | 119.27 | 103.29 | 86.21 | 67.15 |
| C8 | 0.75 | 169.58 | 155.75 | 141.46 | 126.61 | 110.96 | 94.15 | 75.12 |
| C9 | 0.75 | 174.92 | 161.29 | 147.20 | 132.52 | 117.03 | 100.29 | 81.13 |
| C5 | 1 | 136.09 | 121.44 | 106.11 | 89.91 | 72.54 | 53.38 | 31.00 |
| C6 | 1 | 146.62 | 133.09 | 119.03 | 104.30 | 88.73 | 71.94 | 53.38 |

| | | | | | | | | |
|---|---|---|---|---|---|---|---|---|
| C7 | 1 | 154.12 | 141.14 | 127.66 | 113.56 | 98.66 | 82.56 | 64.55 |
| C8 | 1 | 160.08 | 147.43 | 134.31 | 120.55 | 105.99 | 90.15 | 72.22 |
| C9 | 1 | 165.13 | 152.68 | 139.75 | 126.19 | 111.79 | 96.04 | 77.99 |
| C5 | 1.25 | 127.18 | 113.88 | 99.85 | 84.93 | 68.78 | 50.85 | 29.62 |
| C6 | 1.25 | 137.02 | 124.80 | 112.01 | 98.52 | 84.13 | 68.54 | 51.01 |
| C7 | 1.25 | 144.03 | 132.35 | 120.13 | 107.27 | 93.55 | 78.65 | 61.68 |
| C8 | 1.25 | 149.60 | 138.25 | 126.38 | 113.87 | 100.50 | 85.89 | 69.01 |
| C9 | 1.25 | 154.32 | 143.17 | 131.51 | 119.19 | 105.99 | 91.49 | 74.52 |
| C5 | 1.5 | 126.80 | 113.59 | 99.66 | 84.81 | 68.72 | 50.85 | 29.65 |
| C6 | 1.5 | 136.61 | 124.49 | 111.79 | 98.38 | 84.06 | 68.53 | 51.06 |
| C7 | 1.5 | 143.61 | 132.02 | 119.90 | 107.12 | 93.47 | 78.64 | 61.74 |
| C8 | 1.5 | 149.16 | 137.91 | 126.14 | 113.71 | 100.41 | 85.88 | 69.07 |
| C9 | 1.5 | 153.86 | 142.81 | 131.25 | 119.02 | 105.90 | 91.49 | 74.59 |
| C5 | 1.75 | 126.79 | 113.58 | 99.65 | 84.79 | 68.72 | 50.85 | 29.65 |
| C6 | 1.75 | 136.61 | 124.48 | 111.77 | 98.36 | 84.05 | 68.53 | 51.06 |
| C7 | 1.75 | 143.60 | 132.01 | 119.88 | 107.10 | 93.47 | 78.64 | 61.74 |
| C8 | 1.75 | 149.15 | 137.90 | 126.12 | 113.69 | 100.41 | 85.88 | 69.07 |
| C9 | 1.75 | 153.85 | 142.80 | 131.23 | 119.01 | 105.90 | 91.48 | 74.60 |
| C5 | 2 | 153.85 | 114.00 | 99.98 | 85.05 | 68.91 | 50.96 | 29.71 |
| C6 | 2 | 137.14 | 124.93 | 112.15 | 98.66 | 84.28 | 68.69 | 51.16 |
| C7 | 2 | 144.16 | 132.49 | 120.29 | 107.43 | 93.72 | 78.82 | 61.86 |
| C8 | 2 | 149.73 | 138.40 | 126.55 | 114.04 | 100.68 | 86.08 | 69.21 |
| C9 | 2 | 154.45 | 143.32 | 131.68 | 119.37 | 106.19 | 91.69 | 74.74 |
